# The origin point of the unstable solution area of a forced softening Duffing oscillator

**DOI:** 10.1038/s41598-022-07932-8

**Published:** 2022-03-16

**Authors:** Wojciech Wawrzynski

**Affiliations:** Department of Ship Operation, Faculty of Navigation, Gdynia Maritime University, Aleja Jana Pawła II 3, 81-345 Gdynia, Poland

**Keywords:** Mechanical engineering, Nonlinear phenomena

## Abstract

Each Duffing equation has an unstable solution area with a boundary, which is also a line of bifurcation. Generally, in a system that can be modeled by the Duffing equation, bifurcations can occur at frequencies lower than the origin point frequency of the unstable solution area for a softening system and at higher frequencies for a hardening system. The main goal of this research is to determine the analytical formulas for the origin point of the unstable solution area of a system described by a forced Duffing oscillator with softening stiffness, taking damping into account. To achieve this goal, two systems of softening Duffing oscillators that differ strongly in their nonlinearity factor value have been selected and tested. For each system, for three combinations of linear and nonlinear stiffness coefficients with the same nonlinearity factor, bistability areas and unstable solution areas were determined for a series of damping coefficient values. For each case, curves determined for different damping values were grouped to obtain the origin point curve of the unstable solution, ultimately developing the target formulas.

## Introduction

The Duffing equation was introduced by Georg Duffing in^1^ and is considered one of the prototype systems of nonlinear dynamics^2^. The equation is very popular and can be used to model a variety of physical systems, e.g. pendulums^3,4^ beam buckling^5,6^, nonlinear electronic circuits^7,8^, nonlinear vibration isolators^9,10^, flexible link manipulators^11^, and ship rolling^12,13^. Duffing oscillators can also be used to detect local defects, such as those existing on deep groove ball bearing races^14^, pipes^15^ or plate-like structures^16^. Notwithstanding, the most common field of application of the Duffing equation seems to be mechanics, where its basic form is considered a mathematical model of motion of a single degree-of-freedom system, with linear damping and nonlinear stiffness. Obviously, it can also be used to describe one or more degrees of freedom in MDOF systems^17–20^. Generally, the Duffing equation is used to model nonlinear dynamic systems where the state changes exist as oscillations, so it is often called the Duffing oscillator.

Despite the simplicity of the Duffing oscillator, its dynamic behavior is rich, and research on it is still ongoing^2^. The characteristic for nonlinear oscillations, and thus for the Duffing equation, is the resonance frequency dependence on oscillation amplitude. The derivatives of this phenomenon are bifurcations, jumps in oscillation amplitude and bistability, otherwise known as chaotic motion. Therefore, much research is still devoted directly to the Duffing equation^21–24^.

The range of Duffing oscillator applications indicates that the related literature is extremely wide and includes numerous books and many studies published in scientific journals. Searching the ScienceDirect platform with the term Duffing gives over 1000 results for 2020 and 2021. Books devoted to nonlinear systems present a comprehensive description of the issue, covering the knowledge that is already known^2,25–27^, while studies published in scientific journals usually concern a well-defined problem^14,28,29^ or propose a new solution method^30^, new analytical derivations^31,32^ or a new tool that may be useful in the analysis of nonlinear dynamic systems^33^.

The Duffing oscillator in its standard form is a nonlinear second-order differential equation with a constant value of damping and stiffness described by a cubic polynomial. To solve a defined problem, for a system described by this equation, an analytical^32,34,35^ or/and numerical^13,36–38^ approach can be applied. To provide credibility for a mathematical model, experiments may be conducted^21,32,39^. Among analytical methods, the most popular are the harmonic balance method (HBM)^27,40–43^, the iteration method^44–46^, the method of multiple scales^47^, the perturbation method^48^, the homotopy perturbation method^49–51^ and the continuation method^37,52,53^.

When the analytical approach is used, to obtain derivations directly from the Duffing equation, the values of the equation parameters are usually assumed to be constant, the number of parameters is reduced (by transforming the equation), and some additional simplifications are introduced^31,32^. Analytical formulas that are determined in this way are convenient and useful, but they always have limitations, i.e., they are restricted to very small damping values. Moreover, these formulas refer to the steady-state solution, disregarding the process of achieving it and giving steady-state solution even in the area where the equation is unstable.

It is also worth noting that most real nonlinear dynamic systems are more complex than the standard Duffing equation can model. Damping is nonlinear and dependent on amplitude as well as oscillation frequency. Stiffness is given by a polynomial of higher degree than three, the amplitude of the driving force usually changes in time, and some additional external excitation impulses can occur. For these cases, the Duffing equation should be modified into a Duffing-type equation. Naturally, an analysis of a complex system is more problematic, and analytic derivations and generalized conclusions are usually not obtained. In such cases, a group of specialized tools is very useful.

As commonly used tools in the analysis of nonlinear systems, the frequency response curve ^21,27,31–35,39,41,42,46,54,55^, backbone curve^13,31,39^ and time histories^16,30,56^ can be identified. More sophisticated tools include phase portraits^16,24,57–59^, bifurcation diagrams^24,55,59^, basins of attraction^60^ and Poincaré maps^16,59^.

The tool that is most always used in the analysis of nonlinear systems is the frequency response curve. Generally, this tool is used to indicate the resonance frequency, and when the system is nonlinear, jump-up and jump-down frequencies and amplitudes (bifurcations). The problem is that the frequency response curve is commonly determined for a constant value of the excitation amplitude, while as stated earlier, the value of the excitation amplitude varies in time in more complex systems, and time-dependent external and internal disturbances can appear. To obtain a more complete picture of the system, charts of bistability areas and unstable solution areas of nonlinear systems can be used^13,33,56^. For an analyzed system, lines that define the upper and lower limits of the unstable solution areas are the lines of bifurcation; thus, knowledge of the location of this area is important.

The main goal of this research was to determine analytical formulas for the origin point of the unstable solution area of a forced Duffing oscillator with softening stiffness, taking into account damping. To achieve this, two systems of softening Duffing oscillators that differ strongly in the value of the nonlinearity factor have been selected. For each system, for three combinations of equation coefficients but the same nonlinearity factor, bistability areas and area of unstable solution were determined for a series of damping coefficients. For each case, curves determined for different damping values were grouped to obtain the origin point curve of the unstable solution. Finally, analytical formulas for the position of the origin point were developed. Knowing the position of the origin point, it should be possible to determine the analytical formulas for the curves describing bistability area and unstable solution area charts of a nonlinear system modeled by a Duffing equation with any value of damping. It will not be necessary to perform tedious and time-consuming numerical simulations to identify these areas.

## Duffing equation explored in this research

In the present research, the forced Duffing oscillator in almost standard form was explored:

1 $$ \ddot{x} + 2\eta \dot{x} + \alpha x + \beta x^{3} = \xi \left( t \right)cos\left( {\omega t} \right) $$

where *η,* the parameter of the first derivative, controls damping that is linear, *α* and *β* are stiffness (restoring) coefficients, *ξ* is the coefficient of excitation amplitude that works with the angular frequency *ω* and *t* is the time.

The excitation coefficient *ξ* is time-dependent due to the procedure for determining the bistability and unstable solution areas of Eq. (1). For example, to determine the lower limit of the unstable solution area (1), the point where the solution jump-up (bifurcation point) should be revealed at any frequency in this area. To do this, a two-step procedure was used. In the first step, numerical simulations were carried out for the considered spectrum of *ω*, where at each *ω* value, the value of *ξ* was slowly incremented from 0 to the jump-up value. This step provides accurate information about the frequency range of unstable solutions but only an approximate amplitude value at which the solution jumps. To determine the exact value of the jump-up amplitude, in the next step, a procedure for increasing the excitation coefficient was realized according to the sine function for at least the first 1000 s to obtain the smallest possible *ξ* increments near the jump-up point. More information about determining the bistability and unstable solution areas in Eq. (1) can be found in^38^.

The stiffness, which is described by cubic polynomial *α x* + *β x*^3^, has a linear part controlled by *α* and a nonlinear part controlled by *β*. When *α* is > 0, then for *β* < 0, the stiffness characteristic is softening, and for *β* > 0, it is hardening. When *β* = 0, then Eq. (1) describes a simple harmonic oscillator with linear damping (constant value of *η*).

To describe the nonlinearity of the Duffing equation, a nonlinearity coefficient is often used. The form of this coefficient can slightly differ among studies (not only in terms of notation) due to the issue that is considered. However, the essential part of this coefficient always contains the following form:

2 $$ \varepsilon = \frac{\beta }{{\alpha^{3} }} $$

Although the nonlinearity coefficient *ε* is commonly used, it is an ambiguous parameter. This is because we can obtain the same value of *ε* for different combinations of *α* and *β* (Table 1). For the Duffing equation with nonlinear stiffness described by the formula *α x* + *β x*^3^, the crucial parameter is the *α* coefficient, where the *β* coefficient, in the case of a softening system, determines only the value of the critical amplitude of oscillation (the maximum possible amplitude of oscillation)^33^.

**Table 1:** Coefficients of the Duffing equation that were tested during research (softening systems).

Case no	*α*	*β*	${\varvec{\varepsilon}} = \frac{{\varvec{\beta}}}{{{\varvec{\alpha}}^{3} }}$	*Y*_*cr*_	*α·Y*_*cr*_
**Group A**					
A1	1.44225	− 0.0018	− 0.0006	28.30	40.82
A2	1.00000	− 0.0006	− 0.0006	40.82	40.82
A3	0.693361	− 0.0002	− 0.0006	58.87	40.82
**Group B**					
B1	1.44225	− 0.135	− 0.045	3.27	4.71
B2	1.00000	− 0.045	− 0.045	4.71	4.71
B3	0.693361	− 0.015	− 0.045	6.79	4.71

## Oscillation bistability areas and unstable solution areas of the Duffing equation

**Figure 1:**
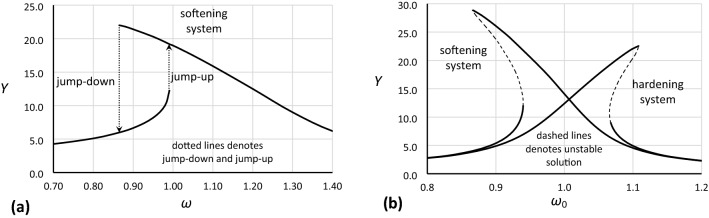
Frequency response curves determined from numerical simulations (**a**) and by analytical solution (**b**).

In the analysis of dynamic nonlinear systems, one of the basic tools is the frequency response curve, as shown in Fig. 1. For a particular system, the frequency response curve can be determined from experiments or numerical simulations (Fig. 1a) or can be calculated with the use of analytical formulas (Fig. 1b). Obviously, experiments can provide results on the highest level of reality, but time and cost constraints often negate using this approach. Of the other two methods, analytical formulas ignore the dynamic process of achieving a steady-state solution and are derived assuming a number of simplifications that in turn result in a stable solution, even in the areas where Eq. (1) is unstable: in Fig. 1b, these curve segments are marked by dashed lines. Additionally, frequency response curves determined by analytical formulas do not directly reveal the points of bifurcation where jump-up and jump-down occur: independent formulas should be derived to show these points. On the other hand, numerical simulations require much more time to determine the frequency response curve, and specialized software must typically be used, even if a computer algebra system (CAS) is used.

**Figure 2:**
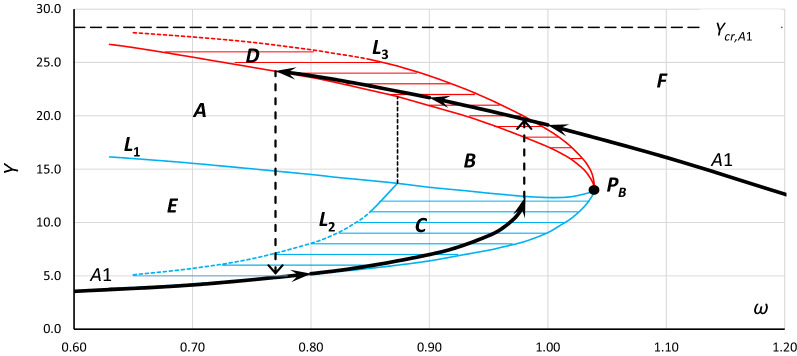
Bistability and unstable solution areas of a Duffing equation for case A1 (Table 1), calculated with the use of Eq. (1) with damping coefficient *η* = 0.10 and frequency response curves for a specific value of excitation coefficient (line A1).

Regardless of the method used to determine the frequency response curve, this curve can be insufficient. The problem concerns systems where the amplitude of excitation can vary in time or some external impulses can occur or when the process of achieving the steady-state solution matters. To analyze such a system, charts of bistability and unstable solution areas are necessary^13,33,56^. An example of a chart showing bistability and unstable solution areas of a Duffing equation for case A1 (Table 1) is presented in Fig. 2. Currently, to determine these charts, numerical simulations or experiments are indispensable.

In brief, the parts of Fig. 2 can be described as follows:

- Point *P*_*B*_ is the bistability origin point. This point is also the origin point of the unstable solution area of the Duffing equation. Bifurcations can occur from this point at lower frequencies for a softening system and at higher frequencies for a hardening system.
- Areas *C* and *D* are oscillation bistability areas. In area C, the oscillations are nonresonant, while in area D, the oscillations are resonant. Transitions between both areas are possible and were discussed in^38^.
- Areas *A* and *B* are unstable solution areas of the Duffing equation. The upper and lower limits of these areas are the lines of bifurcation. Regardless of the parameter that is changing, entering into both areas causes a jump. Entering area *B* at the lower limit causes a jump to the upper limit of area *D,* where entering area *B* at the upper limit causes a jump to the lower limit of area *C*. Area *A* slightly differs from area *B*. The lower limit of this area (line *L*_1_) refers to the excitation amplitude at which the system jumps over the critical amplitude (*Y*_***cr***_).
- Line *L*_1_ is the line of bifurcation/jump-up. In other words, it is the line of the maximum amplitude of nonresonant oscillations that can be obtained with the smooth increase in the value of excitation amplitude. Inside region *A*, a further increase in the excitation amplitude induces a jump-up over *Y*_*cr*_. The same action inside region *B* induces a jump-up to the upper limit of area *D* and above it.
- Line *L*_2_ is the line of maximum amplitude of nonresonant oscillations at which it is possible to force (by additional impulse) a jump to the area of resonant oscillations.

For a comprehensive description of the diagram presented in Fig. 2, refer to^33^.

The frequency response curves calculated for the same coefficient of nonlinearity *ε* are usually scaled/converted to the nondimensional form according to the following rules:

3 $$ \omega_{0} = \frac{\omega }{\sqrt \alpha }\, Y_{0} = \alpha Y $$

The same procedure can be implemented for bistability areas; however, it should be clearly emphasized that rule (3) works only when the damping value is very small, in fact, when damping is close to zero (this also applies to the frequency response curves). In^33^, it was shown that two systems that differ even very strongly in the value of *ε* but have the same values of the *α* coefficient and the same values of the damping coefficient (which do not have to be close to zero) have identical bistability and unstable solution areas when their *Y* values are recalculated according to the following formula:

4 $$ Y_{n} = \frac{Y}{{Y_{cr} }} $$

where *Y*_*cr*_ is the critical value of the oscillation amplitude, which can be calculated by the following formula for the softening system:

5 $$ Y_{cr} = \sqrt {\left| {\frac{\alpha }{\beta }} \right|} $$

Generally, to describe any Duffing oscillator with softening stiffness defined as in Eq. (1), only the *α* coefficient and damping coefficient *η* are needed^33^. Conversion between the charts (Fig. 2) calculated for different values of the *α* coefficient but the same damping is simple. The relationships are defined by formula (6)^33^. It is important to note that for damping coefficients different than zero, there is no way to convert the frequency axis to a nondimensional form.

6 $$ \alpha_{1} \cdot Y_{cr1} = \alpha_{2} \cdot Y_{cr2} $$

In^33^, it was shown that for the standard Duffing Eq. (1), one set of charts (calculated for a series of damping coefficient values) can adequately describe it. This set of drawings can be converted to another set; for any combination of *α* and *β* with only one reservation, the damping coefficients *η* should be the same.

## Numerical simulations and analysis of results

Data used in the analysis were obtained from numerical simulations performed for two groups of coefficients of Duffing Eq. (1), presented in Table 1. These groups differ strongly in the value of the nonlinearity coefficient *ε*; for all cases in Group A, *ε* =  − 0.0006, while for all cases in Group B, *ε* =  − 0.045. Regardless of differences in the value of *ε*, the coefficients of the linear part of the stiffness are the same in both groups to obtain the corresponding cases.

Numerical simulations were performed with the use of CAS programs (*Mathematica* and *MATLAB*) using the Runge–Kutta method and the same initial conditions ($x = 0, \dot{x} = 0$) for a series of damping coefficients: *η* = 0.001, 0.01, 0.02, 0.035, 0.05, 0.075, 0.10, 0.15, 0.20, 0.25, 0.30, and 0.35 (for each case from Table 1). Some simulations were carried out during previous research^33^. Globally, as a result of all the numerical simulations, 72 diagrams, such as the one presented in Fig. 2, were obtained.

**Figure 3:**
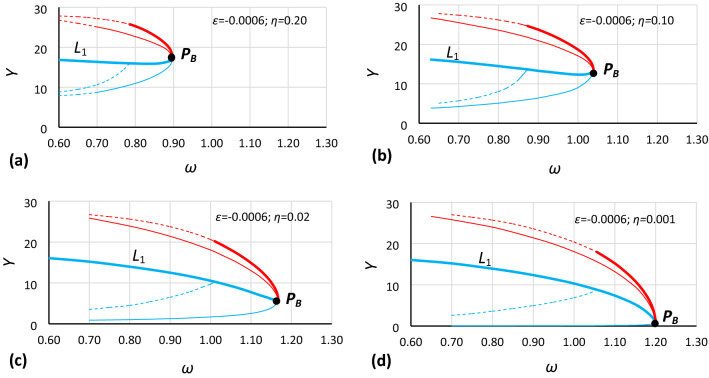
Bistability areas, unstable solution areas and origin point *P*_*B*_ of the Duffing equation for nonlinearity *ε* =  − 0.0006. Case A1 (*α* = 1.44225; *β* =  − 0.0018) for damping coefficient *η*: (**a**) 0.20, (**b**) 0.10, (**c**) 0.02, (**d**) 0.001.

**Figure 4:**
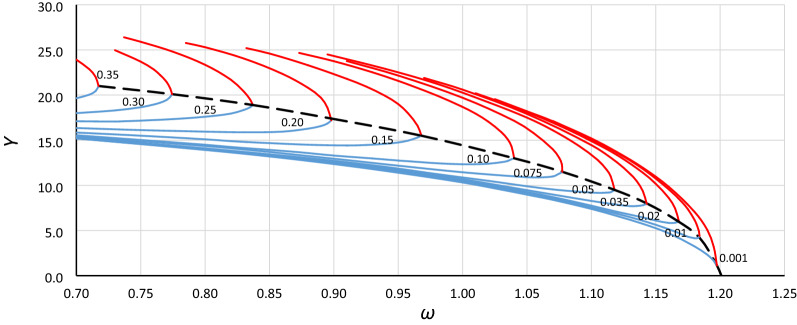
Curve of the origin point *P*_*B*_ for case A1 (*α* = 1.44225, *β* =  − 0.0018, *ε* =  − 0.0006) determined as a curve of convergence points of line *L*_1_ (blue lines) and the upper limit of the bistability area for resonant oscillations (red lines), calculated for different values of damping coefficient *η* (from 0.001 to 0.35).

**Figure 5:**
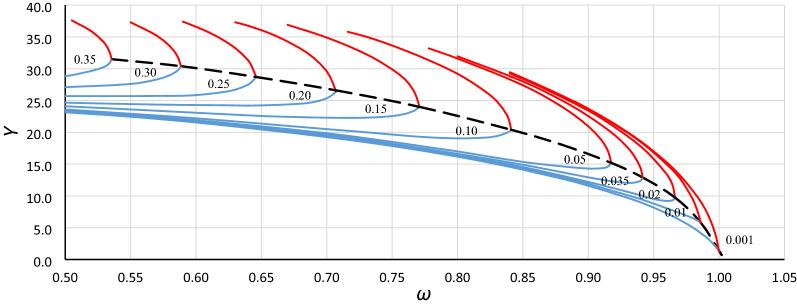
Curve of the origin point *P*_*B*_ for case A2 (*α* = 1.0000, *β* =  − 0.0006, *ε* =  − 0.0006) determined as a curve of convergence points of line *L*_1_ (blue lines) and the upper limit of the bistability area for resonant oscillations (red lines), calculated for different values of damping coefficient *η* (from 0.001 to 0.35).

**Figure 6:**
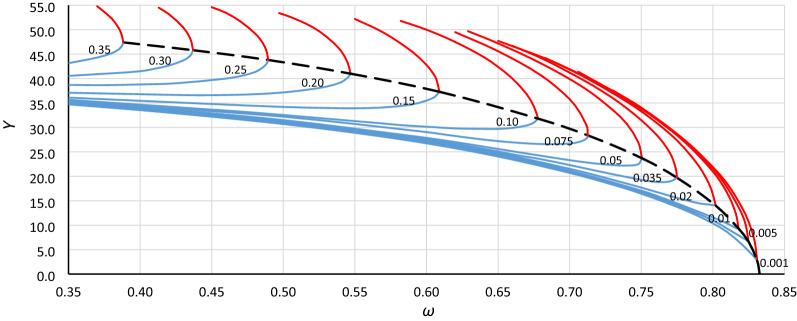
Curve of the origin point *P*_*B*_ for case A3 (*α* = 0.693361, *β* =  − 0.0002, *ε* =  − 0.0006) determined as a curve of convergence points of line *L*_1_ (blue lines) and the upper limit of the bistability area for resonant oscillations (red lines), calculated for different values of damping coefficient *η* (from 0.001 to 0.35).

**Figure 7:**
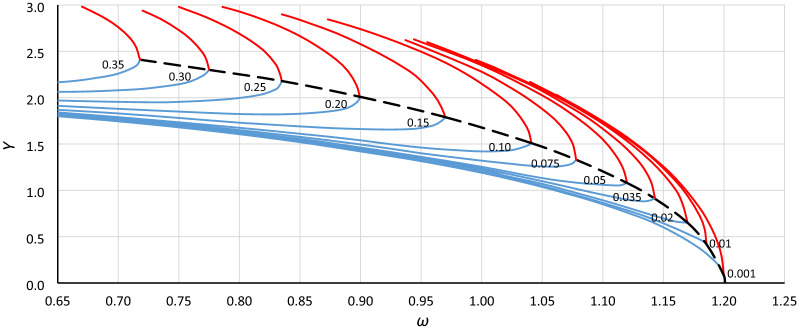
Curve of the origin point *P*_*B*_ for case B1 (*α* = 1.44225, *β* =  − 0.135, *ε* =  − 0.045) determined as a curve of convergence points of line *L*_1_ (blue lines) and the upper limit of the bistability area for resonant oscillations (red lines), calculated for different values of damping coefficient *η* (from 0.001 to 0.35).

**Figure 8:**
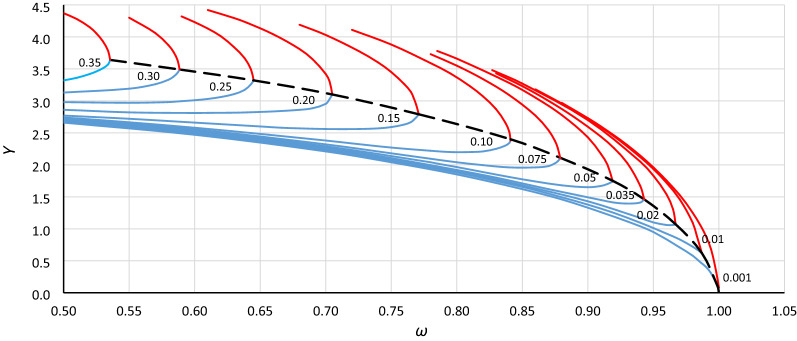
Curve of the origin point *P*_*B*_ for case B2 (*α* = 1.0000, *β* =  − 0.045, *ε* =  − 0.045) determined as a curve of convergence points of line *L*_1_ (blue lines) and the upper limit of the bistability area for resonant oscillations (red lines), calculated for different values of damping coefficient *η* (from 0.001 to 0.35).

**Figure 9:**
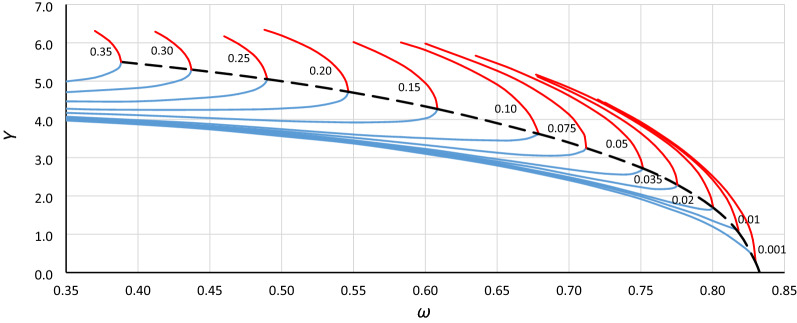
Curve of the origin point *P*_*B*_ for case B3 (*α* = 0.69336, *β* =  − 0.015, *ε* =  − 0.045) determined as a curve of convergence points of line *L*_1_ (blue lines) and the upper limit of the bistability area for resonant oscillations (red lines), calculated for different values of damping coefficient *η* (from 0.001 to 0.35).

To analyze the location of the origin point of the unstable solution area of the Duffing equation (abbreviated hereinafter as *origin point P*_*B*_), the bistability areas for different values of damping have been grouped for each case in Table 1 (as in Fig. 3). Curves of the origin point *P*_*B*_ have been determined by combining the convergence points of line *L*_1_ (Fig. 3 – solid bold blue lines) and the line of the upper limit of the bistability area for resonant oscillations (Fig. 3 – solid bold red lines), and those for cases A1, A2, A3 and B1, B2, B3 are presented in Figs. 4, 5, 6, 7, 8 and 9, respectively.

**Figure 10:**
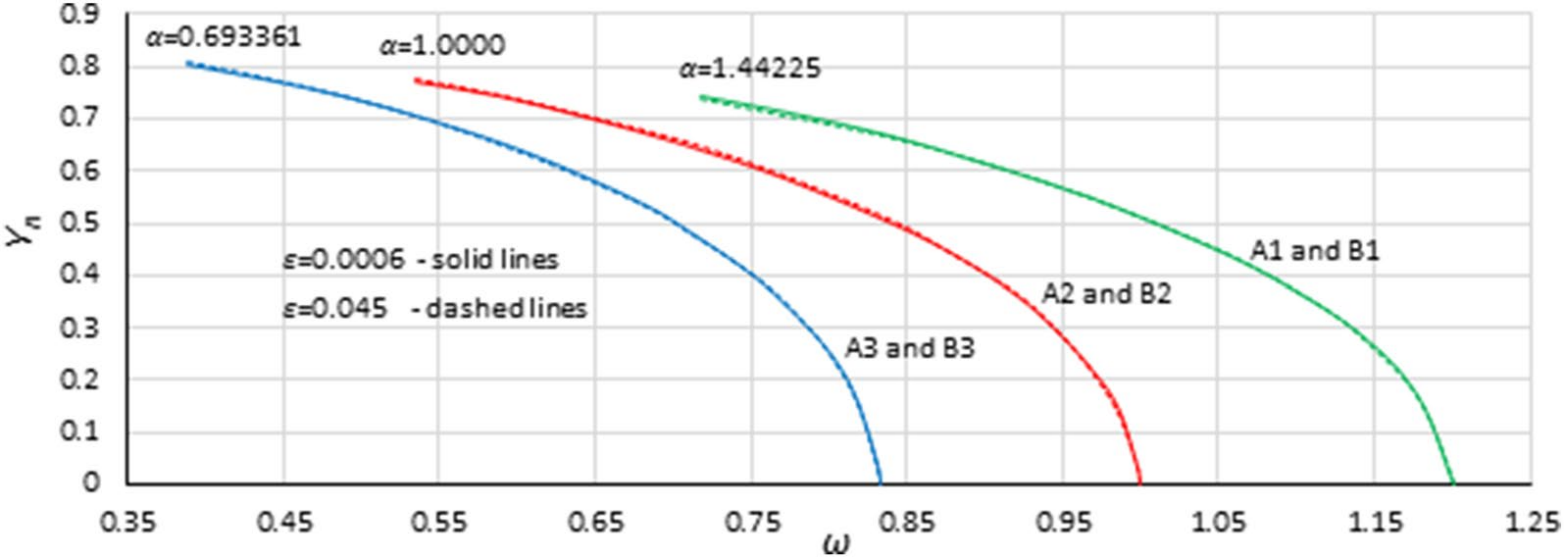
Comparison of curves of the origin point *P*_*B*_ for cases A1 (green solid line), A2 (red solid line), A3 (blue solid line) and B1 (green dashed line), B2 (red dashed line), and B3 (blue dashed line), all recalculated for nondimensional value *Y*_*n*_ according to formula (4).

Curves of the origin point *P*_*B*_ presented in Figs. 4, 5, 6, 7, 8 and 9 look similar. To compare them, oscillation amplitude values were converted to a nondimensional form according to Formula (4). The comparison is shown in Fig. 10. It is clearly seen that curves of the origin point of the unstable solution area of the Duffing equation are almost identical for the same values of *α*, independent of the nonlinearity coefficient value *ε*. Therefore, in the following analysis, averaged curves (A1 with B1, A2 with B2 and A3 with B3) were used.

Although the curves in Fig. 10 were obtained on the basis of simulations made for different damping values, they are independent of damping. This means that curves of the origin point *P*_*B*_ indicate the direct characteristics of the nonlinear system, where damping determines only the point on the curve. The consequence of this fact is that the curves in Fig. 10 should be described by an equation that bypasses damping.

To determine the formula that describes the curve of the origin point, the standard fitting procedures were discarded. This was because formulas obtained in accordance with these methods can fit well in the range of input data (points from numerical simulations) and usually have an unconvincing run beyond them. During the research, numerical simulations were carried out for damping coefficients up to 0.35. For larger *η* values, some results were incredible—the response curves were distorted. However, the course of the *P*_*B*_ curve outside the points obtained on the basis of numerical simulations can be quite easily predicted without any additional calculations. It is clear that the ordinate of the *P*_*B*_ curve for larger values of *η* will tend to *Y*_*cr*_ (Figs. 4, 5, 6, 7, 8, 9) or to 1 for scaled curves (Fig. 10). Therefore, to achieve a reasonable solution, it was necessary to use the following formula: *c*_0_*ω*^p0^ = *c*_1_ + *c*_2_*Y*^p2^ + *c*_3_*Y*^p3^ + …. In this formula, the first two terms of the right side force the general course of the curve, and the terms with higher power can be used to adjust the course of the curve.

Because the location of the origin point *P*_*B*_ is always close to the backbone curve^33^, to determine the formula that describes the curve of the origin point, the simple Formula (7) for the backbone curve (for damping equal to 0) was chosen as a base.

7 $$ \omega_{0;backbone}^{2} = 1 \pm \frac{3}{4}\varepsilon Y^{2} $$

To simplify the task, the base formula was determined for case A2 (this reduces the *α* coefficient to *α* = 1) and the scaled curve (this reduces the *β* coefficient). After these simplifications, during the fitting procedure, the base formula was determined as:

8 $$ \omega^{2} = 1 - \sqrt{\frac{4}{3}}  Y_{n}^{2} - \left( {\frac{1}{2}Y_{n} } \right)^{4} $$

To include the impact of the α coefficient, it is sufficient to multiply the right side of Formula (8) by *α*:

9 $$ \omega^{2} = \alpha \left[ {1 - \sqrt{\frac{4}{3}}  Y_{n}^{2} - \left( {\frac{1}{2}Y_{n} } \right)^{4} } \right] $$

Returning to the curves at the original scale (Figs. 4, 5, 6, 7, 8, 9), we obtain the final form of the formula for the origin point of the unstable solution area of the softening Duffing equation (point *P*_*B*_):

10 $$ \omega^{2} = \alpha - \sqrt{\frac{4}{3}}  \left| \beta \right|Y^{2} - \frac{{\beta^{2} }}{\alpha }\left( {\frac{1}{2}Y} \right)^{4} $$

and taking into account the sign of *β* (softening systems):

11 $$ \omega^{2} = \alpha + \sqrt{\frac{4}{3}}  \beta Y^{2} - \frac{{\beta^{2} }}{\alpha }\left( {\frac{1}{2}Y} \right)^{4} $$

Looking for a simpler solution, it was found that satisfactory results can be obtained using the following formulas:

12 $$ \omega^{2} = \alpha \left( {1 - \frac{19}{{16}}Y_{n}^{2} } \right) $$

13 $$ \omega^{2} = \alpha + \frac{19}{{16}}\beta Y^{2} $$

**Figure 11:**
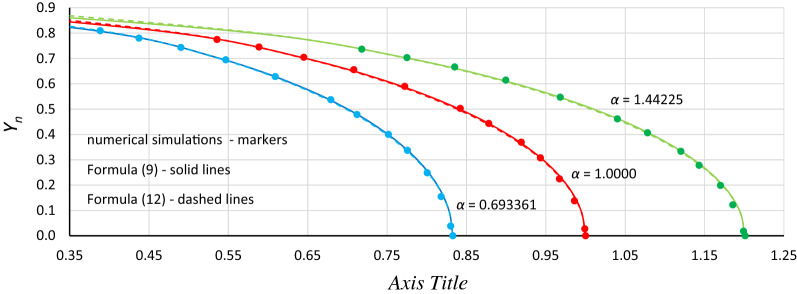
The consistency of analytical formulas (9)—solid line and (12)—dashed line with the results of numerical simulations (markers) for *α* = 0.693361 (blue), *α* = 1.0000 (red) and *α* = 1.44225 (green).

The consistency of formulas (9) and (12) with the results of numerical simulations is shown in Fig. 11. Generally, for each value of *α* that was tested, good coincidence between analytical formulas and numerical simulations was found. Formulas (9) and (12) give almost the same results; however, for *Y*_*n*_ > 0.85, differences became visible. This area is not shown in Fig. 11 because obtaining *Y*_*n*_ > 0.85 requires *η* > 0.40, and numerical simulations were not performed for these damping values. Formula (9) is preferred because it gives better results in the range of mean *Y*_*n*_ values.

**Figure 12:**
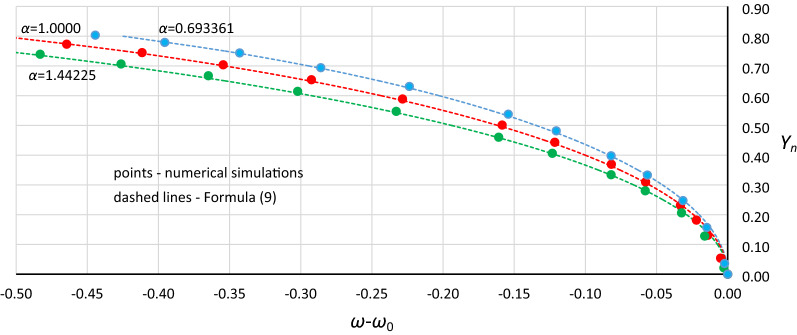
Relative curves of the origin point *P*_*B*_ for *α* = 1.44225 (green line), *α* = 1.0000 (red line) and *α* = 0.693361 (blue line), calculated with formula (9), compared with points of *P*_*B*_ obtained from numerical simulations and recalculated for the nondimensional value *Y*_*n*_.

The next step was to find a relationship between the position of the origin point *P*_*B*_ and damping. Figure 12 shows curves of relative changes in the position of the origin point *P*_*B*_ for different values of the *α* coefficient. These curves were calculated with the use of Formula (9) and show vertical and horizontal displacements of the origin point in relation to point (*ω*_0_ = *α*^0.5^, *Y*_*n*_ = 0). Additionally, the drawing includes points of *P*_*B*_ obtained from numerical simulations, recalculated for the nondimensional value *Y*_*n*_.

**Figure 13:**
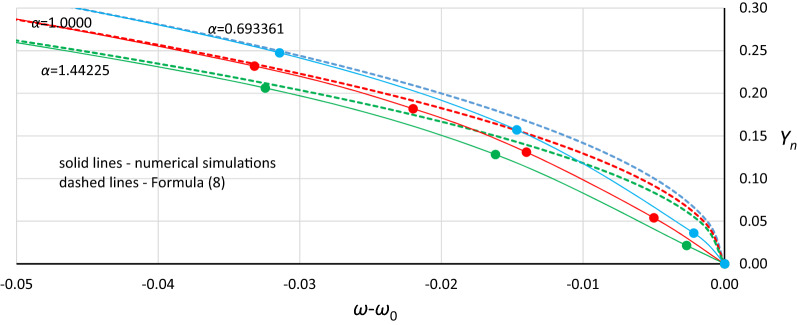
Flattening of relative curves of the origin point *P*_*B*_ obtained on the basis of numerical simulations and the deterioration of coincidence with formula (9) for *Y*_*n*_ ≤ 0.20 (the area of small damping values).

Generally, in Fig. 12, as in Fig. 11, we can observe good coincidence between analytical Formula (9) and the results of numerical simulations. However, in the range of small damping coefficient values, this coincidence was weak in all cases (Fig. 13): for *Y*_*n*_ < 0.20, the damping coefficient *η* ≤ 0.014, while at *Y*_*n*_ ≤ 0.10, the value of *η* goes below 0.005. The problem is more about numerical simulations, where at small damping coefficient values, determining the position of the origin point *P*_*B*_ as a point of convergence of line *L*_1_ and the line of upper limit of the bistability area for resonant oscillations is troublesome. During numerical simulations, the value of *Y* on Line L1 at each frequency was determined for a jump-up (bifurcation point), while the *Y* value of the upper limit of the bistability area for resonant oscillations is the *Y* value of the point to which the solution jumps. The problem is that in the region close to the origin point *P*_*B*_, the amplitude of the mentioned jump is very small and tends to zero at the origin point. Obviously, this problem concerns the curve of *P*_*B*_ as a whole, but for small values of *η*, vertical changes in the curve course are “faster” and more nonlinear (Figs. 4, 5, 6, 7, 8, 9). An additional problem involved observing a jump. Usually, a jump takes no more than a few cycles, and the amplitude changes visibly, while for very small damping values and frequencies close to *P*_*B,*_ the number of cycles can exceed several thousand. The effect of the mentioned problems flattened all the curves obtained on the basis of numerical simulations, as was observed for *Y*_*n*_ ≤ 0.20 (Fig. 13).

**Figure 14:**
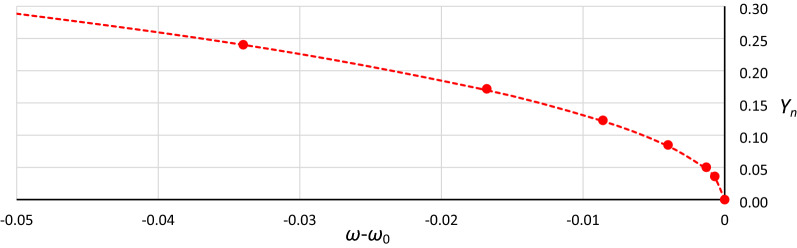
The consistency of the analytical (curve) and numerical (markers) approaches for case B2 (*α* = 1.0000, *β* =  − 0.045, *ε* =  − 0.045) for small values of damping after using the modified procedure for determining the position of *P*_*B*_.

To test whether the above conclusions are correct and whether the analytical formulas that are proposed are effective in the range of small damping values, for one case, the *P*_*B*_ curve was constructed with points of convergence for 4 lines: upper and lower limit lines for both areas of bistability (Figs. 1 and 3). Additionally, the time of each simulation was extended up to 90 000 s, and the solution was considered stable when the oscillation amplitude was constant over a minimum of 10 000 s. The results of using this procedure proved the good consistency of the analytical and numerical approaches, also in the range of small damping values (Fig. 14). However, it should be noted that the application of the procedure took much time.

Considering the effects shown in Fig. 14, it seems justified that in further analysis, for Yn < 0.25, curves calculated according to Formula (9) instead of the data obtained from numerical simulations were used. However, using this approach, the problem is the location of points that correspond to the exact damping value. It was assumed that the connections between frequency and damping, which were determined during the numerical simulations, remained constant, and only the value of *Y*_*n*_ was changed. Additionally, the location of points was adjusted to obtain smooth curve courses.

**Figure 15:**
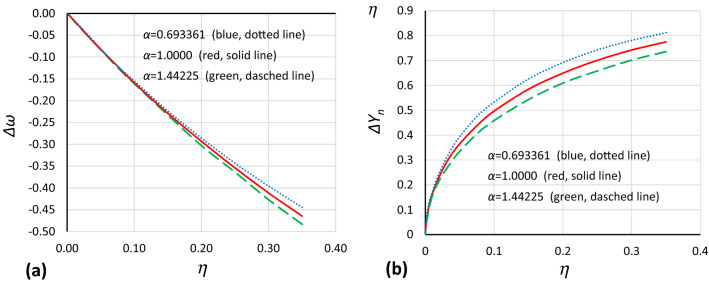
Changes in the position of the origin point *P*_*B*_ due to damping for curves with nondimensional values *Y*_*n*_. Changes in frequency (**a**) and *Y*_*n*_ displacement (**b**).

During the next step, the relative curves of the origin point *P*_*B*_ were split into two curves, each dependent on the damping value: a curve showing changes in frequency (Fig. 15a) and a curve showing *Y*_*n*_ displacement (Fig. 15b).

Using the same fitting procedure as before, it was found that the relationship between damping coefficient *η* and *Y* displacement and between damping coefficient and changes in frequency of the origin point *P*_*B*_ can be described by the following formulas:

14 $$ \eta = 1 - \left[ {1 - \alpha^{1/3} \left( { - \frac{\beta }{\alpha }\Delta Y^{2} + \frac{{\beta^{2} }}{{3\alpha^{2} }}\Delta Y^{4} } \right)} \right]^{1/3} $$

15 $$ \eta = 1 - \left[ {1 - \alpha^{1/3} \left( {\Delta Y_{n}^{2} + \frac{1}{3}\Delta Y_{n}^{4} } \right)} \right]^{1/3} $$

16 $$ \eta = - \sqrt {1/3} \Delta \omega + \frac{3}{{8 \alpha^{{\sqrt {1/3} }} }}\Delta \omega^{2} $$

**Figure 16:**
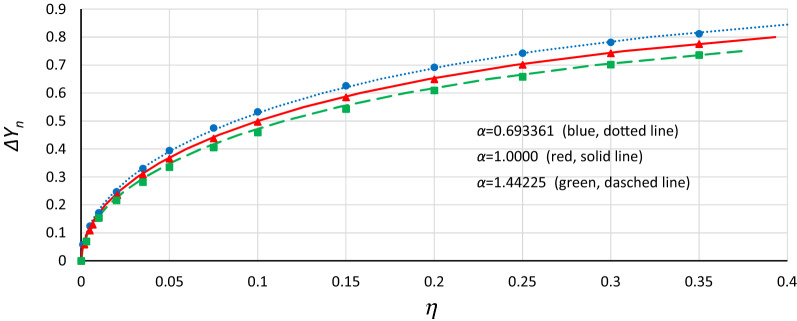
Comparison of the *Y*_*n*_ displacement of the origin point *P*_*B*_ due to damping obtained from numerical simulations (markers) and on the basis of formula (15) (curves).

Figure 16 shows a comparison of the *Y*_*n*_ displacement of the bistability origin point due to damping, determined on the basis of numerical simulations (markers) and obtained with the use of Formula (15) (curves). It is clearly seen that Formula (15) is quite effective.

**Figure 17:**
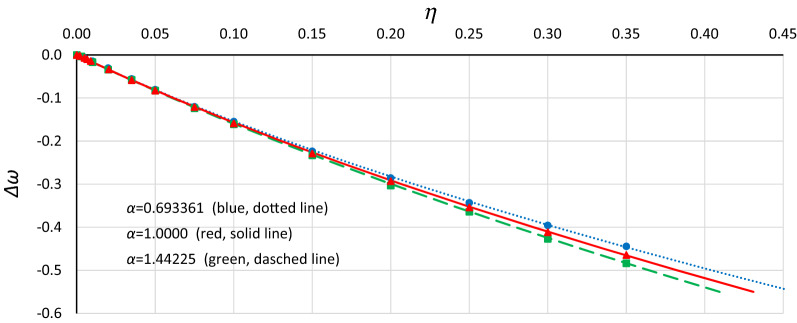
Comparison of the frequency changes of the origin point *P*_*B*_ due to damping obtained from numerical simulations (markers) and on the basis of formula (16) (curves).

A similar effect has been obtained in the case of the frequency changes of the origin point *P*_*B*_ due to damping. In Fig. 17, the comparison of *Δω* determined on the basis of numerical simulations (markers) and obtained from Formula (16) (curves) are shown.

## Conclusions

In this paper, analytical formulas for curves of the origin point *P*_*B*_ of the unstable solution area of the classic Duffing equation with softening stiffness are proposed. Formula (9) concerns curves recalculated for a nondimensional value *Y*_*n*_, while Formula (11) concerns curves in its original scale. It was found that the curve of the origin point *P*_*B*_ has the direct characteristics of a nonlinear system and depends only on the *α* and *β* coefficients (coefficients of the linear and nonlinear parts of the stiffness) or only on *α* when the nondimensional value *Y*_*n*_ is used. In a given system, amplitude jumps (bifurcations) can occur only at frequencies lower than the frequency of *P*_*B*_ for a softening system and at higher frequencies for a hardening system.

Because the curve of the origin point *P*_*B*_ is a direct characteristic of the nonlinear system where the damping value determines only the location of *P*_*B*_ on the curve, formulas on *Y* displacement and the frequency changes of *P*_*B*_ due to damping have also been developed. To calculate the relative displacements of *P*_*B*_, for the original scale of the system, Formulas (14) and (16) should be used, and when *Y* is converted to a nondimensional form *Y*_*n*_, Formulas (15) and (16) should be used. When damping is larger than zero, there is no way to convert the frequency axis to a nondimensional form^33^.

Preliminary analysis performed for a single hardening system showed that Formula (9) with slight modifications may be useful, but the relationship between the curve of the origin point *P*_*B*_ and damping for softening and hardening systems varies considerably, so Formulas (14), (15) and (16) are unusable: hardening systems require independent analysis. Moreover, for hardening systems, the problem of the nondimensional form of the *Y* value should also be analyzed.

Knowing the position of the origin point, it should be possible to determine the analytical formulas for the curves that chart the bistability and unstable solution areas of the nonlinear system described by the Duffing equation with any constant damping value. To determine them, it is unnecessary to perform tedious and time-consuming numerical simulations.
